# 携带胚系突变的骨髓增生异常肿瘤患者临床特征分析

**DOI:** 10.3760/cma.j.cn121090-20250109-00021

**Published:** 2025-06

**Authors:** 晓珍 刘, 喻堤 张, 灵旭 蒋, 琛 梅, 丽 叶, 丽亚 马, 歆平 周, 红艳 佟

**Affiliations:** 浙江大学医学院附属第一医院血液科，杭州 310003 Department of Hematology, the First Affiliated Hospital, Zhejiang University School of Medicine, Hangzhou 310003, China

**Keywords:** 骨髓增生异常肿瘤, 胚系突变, DDX41, Myelodysplastic neoplasm, Germline mutations, DDX41

## Abstract

**目的:**

研究携带胚系突变的骨髓增生异常肿瘤（MDS）患者的临床特征，并探讨胚系突变的预后评估价值。

**方法:**

收集2021年1月至2024年9月浙江大学医学院附属第一医院确诊的407例MDS患者的临床资料，男252例，女155例，中位年龄64（19～85）岁；分析携带胚系突变患者的临床特征，并探索胚系突变的预后评估价值。

**结果:**

MDS患者中胚系突变检出率为5.9％（24/407），在21～30岁年龄段患者中检出率最高（20.0％）。检出的胚系突变基因包括：DDX41 9例（2.2％），TP53 3例（0.7％），RUNX1、TET2、MPL、CBL、ATRX、CEBPA、ETV6、IDH1、KDM5C、SBDS、GNAS、CTC1各1例。与未携带胚系突变患者相比，携带胚系突变患者突出表现为外周血WBC更低（1.87×10^9^/L对2.50×10^9^/L，*P*＝0.018），两者总生存期差异无统计学意义（21.3个月对21.1个月，*P*＝0.97）。相较于携带其他胚系突变的患者，携带DDX41胚系突变的患者中位年龄更大（65岁对54岁，*P*＝0.010），WBC更低（1.51×10^9^/L对2.31×10^9^/L，*P*＝0.040），平均红细胞体积更大（111.80 fl对97.25 fl，*P*＝0.003），正常核型比例更高（100.0％对53.3％，*P*＝0.022）。携带DDX41胚系突变患者最常见合并的体细胞突变为ASXL1、TET2及RUNX1。

**结论:**

本研究中MDS患者胚系突变检出率为5.9％，以21～30岁年龄段检出率最高。最常见的胚系突变基因为DDX41和TP53。携带DDX41胚系突变的MDS患者有相对独特的临床特征。是否携带胚系突变与预后未见相关性。

## Abstract (en)

**Objective:**

To investigate the clinical characteristics and prognostic significance of germline mutations in patients with myelodysplastic neoplasms（MDS）.

**Methods:**

Clinical data from 407 patients with MDS［male, 252; female, 155; median age, 64（range, 19–85）years］diagnosed at the First Affiliated Hospital of Zhejiang University School of Medicine were retrospectively analyzed. The clinical features and prognostic effects of germline mutations were evaluated.

**Results:**

The prevalence of germline mutations in patients with MDS was 5.9％（24/407）, peaking at 20.0％ in the group aged 21–30 years. The spectrum of germline mutations comprised DDX41（9 cases, 2.2％）, TP53（3 cases, 0.7％）, and single cases of RUNX1, TET2, MPL, CBL, ATRX, CEBPA, ETV6, IDH1, KDM5C, SBDS, GNAS, and CTC1. Patients with germline mutations exhibited significantly lower peripheral WBC counts than those without（1.87×10⁹/L *vs* 2.50×10⁹/L, *P*＝0.018）, but showed comparable median overall survival（21.3 months *vs* 21.1 months, *P*＝0.97）. Patients with DDX41 germline mutations, compared with those with other germline mutations, had a significantly older median age（65 *vs* 54 years, *P*＝0.010）, lower WBC counts（1.51×10⁹/L *vs* 2.31×10⁹/L, *P*＝0.040）, increased mean corpuscular volume（111.80 fl *vs* 97.25 fl, *P*＝0.003）, and a higher prevalence of normal karyotypes（100.0％ *vs* 53.3％, *P*＝0.022）. The most frequently co-occurring somatic mutations in DDX41 germline mutation carriers were ASXL1, TET2, and RUNX1.

**Conclusion:**

In this study, the detection rate of germline mutations in MDS patients was 5.9％（24/407）, peaking at 20％ in the group aged 21–30 years. DDX41 and TP53 were the most prevalent germline mutations. DDX41 mutation carriers displayed distinct clinical characteristics; however, germline mutations overall showed no significant prognostic effect.

骨髓增生异常肿瘤（Myelodysplastic neoplasm, MDS），又称骨髓增生异常综合征，是一种起源于造血干/祖细胞的克隆性疾病，其特征是骨髓无效造血、难治性血细胞减少、高风险向急性髓系白血病（AML）转化。MDS好发于老年人，中位年龄为70～75岁[1]–[2]。儿童和年轻人的MDS常常与遗传易感性相关[3]–[4]。为了强调胚系基因评估的重要性，《WHO造血及淋巴组织肿瘤分类（第5版）》将遗传易感倾向的髓系肿瘤列为独立疾病分类[5]。髓系肿瘤患者中胚系突变检出率为5％～15％[4],[6]。一项大型回顾性研究显示，在全年龄段MDS患者中胚系基因突变检出率为7％，其中11～20岁年龄段检出率最高，达到33％[7]。目前我国尚无关于携带胚系突变MDS患者的大样本研究，本研究回顾了单中心携带胚系突变MDS患者的临床特征，现报道如下。

## 病例与方法

一、病例

纳入了2021年1月至2024年9月在浙江大学医学院附属第一医院确诊的407例MDS患者，依据WHO（2016）标准[8]诊断分型，采用修订版国际预后积分系统（IPSS-R）[9]、纳入分子遗传学的国际预后积分系统（IPSS-M）[10]进行风险分层。

二、染色体核型分析

骨髓细胞经过24 h培养，收集细胞常规制片，对至少20个骨髓细胞的中期分裂象采用R显带法分析核型，根据《人类细胞遗传学国际命名体制（ISCN2013）》[11]描述核型，按照IPSS-R[9]对染色体核型进行预后分组。

三、基因检测

从骨髓液中分离单个核细胞，提取DNA，采用多重PCR扩增构建样本库进行高通量测序，参照COSMIC数据库进行生物信息学分析，确定致病基因突变位点。

采用初诊时的口腔上皮细胞作为配对样本进行胚系检测。我院二代测序检测涵盖的髓系肿瘤遗传易感基因包括：ANKRD26、ATRX、BLM、CBL、CEBPA、CTC1、DDX41、DKC1、ELANE、ETV6、FANCA、FANCC、GATA1、GATA2、GFI1、GNAS、HAX1、IDH1、KDM5C、KRAS、MLH1、MSH6、NF1、POT1、PTPN11、RTEL1、RUNX1、SBDS、SRP72、TET2、TERC、TERT、TP53。根据2015年美国医学遗传和基因组学会和分子病理学学会（ACMG/AMP）发布的遗传变异分类标准与指南[12]，胚系突变分为“致病的（P）”“可能致病的（LP）”“意义不明确的（VUS）”“可能良性的（LB）”和“良性的（B）”五个级别。本研究纳入致病的（P）和可能致病的（LP）胚系突变进行分析。

四、随访

采用查阅门诊病历、住院病历或电话联系的方式进行随访，随访截止日期为2024年10月31日。中位随访期为20.7（95％*CI*：18.7～27.9）个月，共35例（8.6％）患者失访。总生存（Overall survival，OS）期定义为诊断日期至死亡日期或随访截止日期。

五、统计学处理

计量资料以中位数（范围）表示，并使用Mann-Whitney *U*检验进行组间比较。计数资料表示为例数（构成比）表示，并通过卡方检验或Fisher精确概率法进行组间比较。采用Kaplan-Meier法绘制生存曲线。双侧*P*<0.05被认为差异有统计学意义。所有分析通过SPSS 26.0、GraphPad Prism 9.0进行。

## 结果

一、患者临床特征

407例MDS患者中男252例，女155例，中位年龄64（19～85）岁。其中，24例（5.9％）患者携带胚系基因突变。临床特征如表1所示，携带胚系突变的MDS患者诊断中位年龄为64（25～84）岁，其中21～30岁年龄段患者胚系突变检出率最高（20.0％）。与未携带胚系突变患者相比，携带胚系突变患者WBC更低［1.87（0.21～8.85）×10^9^/L对2.50（0.35～24.27）×10^9^/L，*P*＝0.018］。其他实验室指标、WHO2016分型、IPSS-R风险分层、IPSS-M风险分层、染色体核型、AML转化率差异均无统计学意义。

**表1:** 407例骨髓增生异常肿瘤（MDS）患者临床特征

指标	携带胚系突变组（24例）	未携带胚系突变组（383例）	*P*值
年龄［岁，*M*（范围）］	64（25～84）	64（19～85）	0.164
性别（例，男/女）	16/8	236/147	0.931
WBC［×10^9^/L，*M*（范围）］	1.87（0.21～8.85）	2.50（0.35～24.27）	0.018
ANC［×10^9^/L，*M*（范围）］	0.85（0.12～3.33）	1.18（0.03～11.96）	0.127
Mono［×10^9^/L，*M*（范围）］	0.10（0.03～0.78）	0.27（0.31～2.24）	0.366
HGB［g/L，*M*（范围）］	70.5（43～120）	75（33～175）	0.359
MCV［fl，*M*（范围）］	105.2（81.5～121.8）	101.7（74.1～136.7）	0.748
PLT［×10^9^/L，*M*（范围）］	40（10～368）	65.5（2～695）	0.076
骨髓原始细胞比例［％，*M*（范围）］	5（0～18）	6（0～18）	0.959
WHO2016分型［例（％）］			0.871
MDS-SLD	4（16.7）	54（14.1）	
MDS-MLD	6（25.0）	63（16.4）	
MDS-RS	1（4.2）	16（4.2）	
MDS伴单纯del（5q）	0	2（0.5）	
MDS-EB-1	6（25.0）	123（32.1）	
MDS-EB-2	7（29.2）	115（30.0）	
MDS-U	0	10（2.6）	
IPSS-R风险分层［例（％）］			0.241
极低危	0	12（3.1）	
低危	4（16.7）	56（14.6）	
中危	7（29.2）	90（23.5）	
高危	2（8.3）	101（26.4）	
极高危	11（45.8）	124（32.4）	
IPSS-M风险分层［例（％）］			0.832
极低危	0	5（1.3）	
低危	2（8.3）	50（13.1）	
中低危	3（12.5）	31（8.1）	
中高危	2（8.3）	57（14.9）	
高危	6（25.0）	96（25.1）	
极高危	11（45.8）	144（37.6）	
染色体核型［例（％）］			
正常核型	15（62.5）	184（48.0）	0.169
复杂核型	5（20.8）	83（21.7）	1.000
AML转化率［例（％）］	4（16.7）	62（16.2）	1.000

**注** Mono：单核细胞计数；MCV：平均红细胞体积；MDS-SLD：MDS伴单系血细胞发育异常；MDS-MLD：MDS伴多系血细胞发育异常；MDS-RS：MDS伴环状铁粒幼红细胞增多；MDS-EB-1：MDS伴原始细胞增多-1型；MDS-EB-2：MDS伴原始细胞增多-2型；MDS-U：MDS不能分类；IPSS-R：修订国际预后积分系统；IPSS-M：纳入分子遗传学的国际预后积分系统；AML：急性髓系白血病

二、胚系基因突变情况

共24例MDS患者检测到致病性或可疑致病性胚系突变，患者胚系基因及体细胞突变信息如表2所示。累及基因包括DDX41 9例，TP53 3例，RUNX1、TET2、MPL、CBL、ATRX、CEBPA、ETV6、IDH1、KDM5C、SBDS、GNAS、CTC1各1例。

**表2:** 24例携带胚系突变骨髓增生异常肿瘤（MDS）患者胚系基因及体细胞突变信息

编号	年龄（岁）	性别	诊断	胚系突变	核苷酸改变	氨基酸改变	体细胞共突变
1	25	男	MDS-EB-1	CTC1	c.374A>G	p.E125G	ASXL1、RUNX1、U2AF1
2	64	男	MDS-MLD	DDX41	c.776A>G	p.Y259C	ASXL1
3	51	女	MDS-SLD	MPL	c.413delT	p.I138fs	PPM1D、BCOR、SRCAP
4	66	男	MDS-SLD	CBL	c.1243G>A	p.G415S	CBL、U2AF1
5	68	女	MDS-EB-2	RUNX1	c.601C>T	p.201X	DNMT3A、SF3B1
6	26	男	MDS-EB-2	SBDS	c.183_184delinsCT	p.K62*	U2AF1、MPL、KRAS
7	68	男	MDS-EB-1	CEBPA	c.365G>A	p.G122E	TP53
8	59	男	MDS-SLD	DDX41	c.776A>G	p.Y259C	DDX41
9	51	女	MDS-MLD	KDM5C	c.2158delG	p.A720fs	JAK2
10	36	女	MDS-EB-1	ATRX	c.3646A>G	p.I1216V	TET2、PHF6
11	36	女	MDS-MLD	TET2	c.4714C>T	p.R1572W	TP53
12	65	女	MDS-EB-2	TP53	c.358A>G	p.K120E	无
13	64	男	MDS-RS-SLD	IDH1	c.297A>G	p.I99M	TET2、SRSF2
14	65	男	MDS-EB-1	DDX41	c.776A>G	p.Y259C	ASXL1、DDX41
15	54	男	MDS-EB-1	ETV6	c.1186A>G	p.R396G	ASXL1、RUNX1、U2AF1、MPL、KDM6A、SETBP1
16	52	女	MDS-MLD	TP53	c.91G>A	p.V31I	DNMT3A
17	64	男	MDS-EB-2	GNAS	c.611C>G	p.A204G	PPM1D、GATA2、SF3B1
18	64	男	MDS-EB-2	DDX41	c.776A>G	p.Y259C	DDX41、TET2、GATA2、STAT3
19	84	女	MDS-SLD	DDX41	c.773C>T	p.P258L	DDX41
20	55	男	MDS-MLD	TP53	c.91G>A	p.V31I	DNMT3A、CUX1、LYST、TNFAIP3
21	60	男	MDS-EB-2	DDX41	c.718A>G	p.I240V	ASXL1、RUNX1
22	71	男	MDS-EB-2	DDX41	c.773C>T	p.P258L	ASXL1、DDX41、DNMT3A、TP53、PPM1D
23	72	男	MDS-EB-1	DDX41	c.1496dupC	p.A500fs	DDX41、RUNX1
24	69	男	MDS-MLD	DDX41	c.776A>G	p.Y259C	DDX41、TET2、EZH2

**注** MDS-EB-1：MDS伴原始细胞增多-1型；MDS-MLD：MDS伴多系血细胞发育异常；MDS-SLD：MDS伴单系血细胞发育异常；MDS-EB-2：MDS伴原始细胞增多-2型；MDS-RS-SLD：MDS伴环状铁粒幼红细胞单系血细胞发育异常

三、生存分析

携带胚系突变患者的中位OS期为21.3（95％*CI*：13.4～29.3）个月，未携带胚系突变患者的中位OS期为21.1（95％*CI*：16.8～25.4）个月，两组患者OS期差异无统计学意义（*P*＝0.97）（图1）。携带胚系DDX41突变患者的中位OS期为21.4（95％*CI*：17.3～41.5）个月，未携带胚系DDX41突变患者的中位OS期为20.2（95％*CI*：16.3～24.1）个月，两组患者OS期差异无统计学意义（*P*＝0.54）。与携带胚系DDX41突变患者相比，携带TP53胚系突变患者生存期更短（*P*＝0.059），中位OS期为9.6（95％*CI*：5.5～13.8）个月。

**图1:**
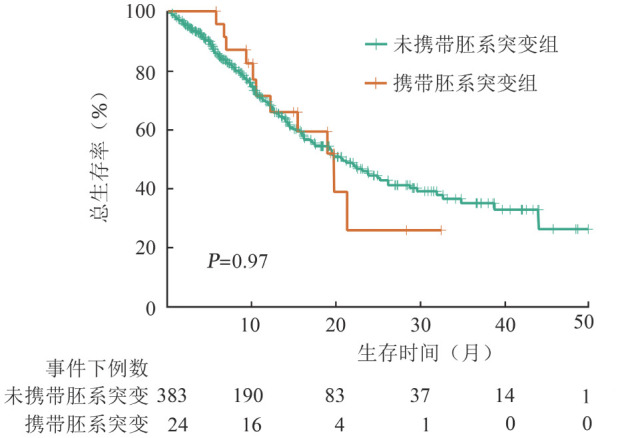
胚系突变对骨髓增生异常肿瘤（MDS）患者总生存期的影响

四、DDX41基因胚系突变

9例携带胚系DDX41突变的患者突变位点如图2所示，包括5例p.Y259C、2例p.P258L、1例p.I240V、1例p.A500fs。其中6例患者同时携带单等位体细胞DDX41突变，1例患者同时携带双等位体细胞DDX41突变。体细胞DDX41突变最常见的位点是p.R525H。

**图2:**
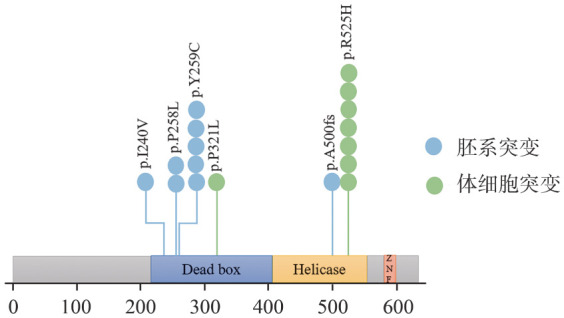
携带胚系DDX41突变骨髓增生异常肿瘤患者突变位点示意图

携带胚系DDX41突变的9例患者的临床特征如表3所示，男性占88.9％（8/9），全部患者均为正常核型，骨髓纤维化分级均为0/1级，5例患者诊断为MDS伴原始细胞增多（MDS-EB），1例患者发生AML转化。与携带其他胚系突变患者相比，携带胚系DDX41突变患者中位年龄更大（65岁对54岁，*P*＝0.010），WBC更低（1.51×10^9^/L对2.31×10^9^/L，*P*＝0.040），平均红细胞体积更大（111.8 fl对97.25 fl，*P*＝0.003），正常核型比例更高（100.0％对53.3％，*P*＝0.022）。

**表3:** 携带胚系DDX41突变与携带其他胚系突变的骨髓增生异常肿瘤（MDS）患者临床特征比较

指标	携带胚系DDX41突变（9例）	携带其他胚系突变（15例）	*P*值
年龄［岁，*M*（范围）］	65（59～84）	54（25～68）	0.010
性别（例，男/女）	8/1	8/7	0.178
WBC［×10^9^/L，*M*（范围）］	1.51（0.74～4.09）	2.31（0.21～8.85）	0.040
ANC［×10^9^/L，*M*（范围）］	0.73（0.20～3.33）	1.01（0.12～2.52）	0.357
Mono［×10^9^/L，*M*（范围）］	0.12（0.04～0.47）	0.36（0.03～0.78）	0.158
HGB［g/L，*M*（范围）］	77（48～106）	64（43～120）	0.858
MCV［fl，*M*（范围）］	111.8（98.5～121.8）	97.25（81.5～118.7）	0.003
PLT［×10^9^/L，*M*（范围）］	26（12～127）	45（10～368）	0.245
骨髓原始细胞比例［％，*M*（范围）］	5（1～15）	7（0～18）	0.881
WHO2016分型［例（％）］			0.903
MDS-SLD	2（22.2）	2（13.3）	
MDS-MLD	2（22.2）	4（26.7）	
MDS-RS	0	1（6.7）	
MDS-EB-1	2（22.2）	4（26.7）	
MDS-EB-2	3（33.3）	4（26.7）	
IPSS-R风险分层［例（％）］			0.273
低危	2（22.2）	2（13.3）	
中危	2（22.2）	5（33.3）	
高危	2（22.2）	0	
极高危	3（33.3）	8（53.3）	
IPSS-M风险分层［例（％）］			0.776
低危	1（11.1）	1（6.7）	
中低危	2（22.2）	1（6.7）	
中高危	1（11.1）	1（6.7）	
高危	2（22.2）	4（26.7）	
极高危	3（33.3）	8（53.3）	
正常核型［例（％）］	9（100.0）	8（53.3）	0.022
AML转化率［例（％）］	1（11.1）	3（20.0）	1.000

**注** Mono：单核细胞计数；MCV：平均红细胞体积；MDS-SLD：MDS伴单系血细胞发育异常；MDS-MLD：MDS伴多系血细胞发育异常；MDS-RS：MDS伴环状铁粒幼红细胞增多；MDS-EB-1：MDS伴原始细胞增多-1型；MDS-EB-2：MDS伴原始细胞增多-2型；IPSS-R：修订国际预后积分系统；IPSS-M：纳入分子遗传学的国际预后积分系统；AML：急性髓系白血病

## 讨论

MDS存在遗传易感性，基因胚系突变增加肿瘤易感性，合并体细胞基因突变二次打击促进肿瘤发生[13]–[14]。2024年NCCN指南中髓系肿瘤遗传易感基因增至30余种[15]。MDS患者携带胚系突变频率为5％～15％[4],[6]–[7],[13]，胚系突变与种族相关[3]。本研究结果显示，MDS患者携带胚系基因突变检出率为5.9％（24/407），在21～30岁年龄段突变检出率最高（20％）。与未携带胚系突变患者相比，携带胚系突变患者除WBC更低（*P*＝0.018）之外，其他临床特征差异无统计学意义。生存分析示携带胚系突变与未携带胚系突变患者的中位OS期差异无统计学意义（21.3个月对21.1个月，*P*＝0.97）。

本研究中，MDS患者最常见的胚系突变累及基因为DDX41。多项研究报道，3％～5％的MDS患者携带DDX41胚系突变，与携带其他胚系突变患者相比不同，携带胚系DDX41突变患者发病年龄更大，易合并血细胞减少[16]–[21]。本组数据显示，DDX41胚系突变检出率为2.2％（9/407），携带DDX41胚系突变的患者较携带其他胚系突变患者年龄更大（65岁对54岁，*P*＝0.010），WBC更低（1.51×10^9^/L对2.31×10^9^/L，*P*＝0.040）。Quesada等[18]报道DDX41胚系突变好发于男性，男女占比为3∶1，且常为正常核型、高级别髓系肿瘤。本组9例DDX41胚系突变患者中，均为正常核型，8例（88.9％）为男性，5例（55.6％）诊断为MDS-EB，与既往报道相符。

DDX41常见胚系突变位点在亚洲人群与西方人群不同。在欧洲人群中常见的突变位点为p.D140fs[22]。在亚洲人群中，Qu等[23]研究中常见的位点包括c.935+4A>T、p.T360Ifs、p.V152G、p.S217Ifs、p.R311*和p.R369*。此外也有报道p.A500fs是亚洲人群特有的位点[22]。本组患者中最常见的突变位点为p.Y259C（5/9，55.6％），1例患者为p.A500fs突变。携带胚系DDX41突变的MDS患者常合并DDX41的体细胞二次打击[10],[17],[22]，本组患者中有7例（77.8％）患者同时携带体细胞DDX41突变，最常见的位点是p.R525H，与Qu等[23]的研究一致。另外，Quesada等[18]的研究显示在胚系DDX41突变的MDS和AML患者中，常常继发TP53突变，但本组患者中最常合并的体细胞突变为ASXL1、TET2、RUNX1。

既往多项研究提示携带DDX41突变的MDS/AML患者预后良好[10],[20]–[21],[23]–[26]，其中Sébert等[21]的研究显示DDX41突变MDS/AML患者接受阿扎胞苷治疗的总缓解率为73％，中位OS期为5.2年，而Makishima等[26]的研究示携带DDX41突变的患者接受去甲基化药物治疗后OS期明显延长（*P*<0.001）。因此，我们推测携带DDX41突变患者的良好预后可能与去甲基化药物治疗有效相关，未来需要在大样本随机临床试验中进一步研究。本组患者中，与未携带DDX41胚系突变患者相比，携带DDX41胚系突变的MDS患者中位OS期无统计学差异［21.4（95％*CI*：17.3～41.5）个月对20.2（95％*CI*：16.3～24.1）个月］。

最近的髓系肿瘤和急性白血病的国际分类共识（ICC2022）[27]、造血和淋巴组织肿瘤第5版WHO（WHO2022）髓系肿瘤分类[5]以及欧洲白血病网对成人AML的诊断和治疗建议[28]均认为DDX41相关髓系肿瘤可能是一个独特亚型，北欧髓系肿瘤胚系易感性工作组在此基础上建议所有髓系肿瘤基因筛查纳入DDX41分析，并进行基线血液学评估和专业遗传咨询[29]。

TP53是第二个相对常见的胚系突变基因。Bougeard等[30]的研究显示胚系TP53突变常与Li-Fraumeni综合征有关，此类患者易患血液系统恶性肿瘤，且预后不良。本研究中携带TP53胚系突变患者仅3例，中位OS期为9.6（95％*CI*：5.5～13.8）个月。TP53胚系突变患者的临床特征需要进一步观察。

进行胚系突变检测对MDS患者的诊疗决策具有重要意义，有利于识别疾病特殊类型，指导疾病危险预后分层及造血干细胞移植供者选择。本组数据显示，MDS患者胚系突变检出率为5.9％，多见于21～30岁患者。DDX41和TP53是最常见的胚系突变基因，是否合并胚系突变与MDS患者的OS未见明显相关性。本研究为单中心回顾性研究，结论有待全国多中心、大样本、前瞻性的研究结果进行证实。

## References

[1] Jaiswal S, Fontanillas P, Flannick J (2014). Age-related clonal hematopoiesis associated with adverse outcomes[J]. N Engl J Med.

[2] Cazzola M (2020). Myelodysplastic Syndromes[J]. N Engl J Med.

[3] Bannon SA, DiNardo CD (2016). Hereditary Predispositions to Myelodysplastic Syndrome[J]. Int J Mol Sci.

[4] Kennedy AL, Shimamura A (2019). Genetic predisposition to MDS: clinical features and clonal evolution[J]. Blood.

[5] Khoury JD, Solary E, Abla O (2022). The 5th edition of the World Health Organization Classification of Haematolymphoid Tumours: Myeloid and Histiocytic/Dendritic Neoplasms[J]. Leukemia.

[6] Klco JM, Mullighan CG (2021). Advances in germline predisposition to acute leukaemias and myeloid neoplasms[J]. Nat Rev Cancer.

[7] Feurstein S, Trottier AM, Estrada-Merly N (2022). Germ line predisposition variants occur in myelodysplastic syndrome patients of all ages[J]. Blood.

[8] Arber DA, Orazi A, Hasserjian R (2016). The 2016 revision to the World Health Organization classification of myeloid neoplasms and acute leukemia[J]. Blood.

[9] Greenberg PL, Tuechler H, Schanz J (2012). Revised international prognostic scoring system for myelodysplastic syndromes[J]. Blood.

[10] Bernard E, Tuechler H, Greenberg PL (2022). Molecular International Prognostic Scoring System for Myelodysplastic Syndromes[J]. NEJM Evid.

[11] Shaffer LG, McGowan-Jordan J, Schmid M (2013). ISCN 2013: An International System for Human Cytogenetic Nomenclature[M].

[12] Richards S, Aziz N, Bale S (2015). Standards and guidelines for the interpretation of sequence variants: a joint consensus recommendation of the American College of Medical Genetics and Genomics and the Association for Molecular Pathology[J]. Genet Med.

[13] Keel SB, Scott A, Sanchez-Bonilla M (2016). Genetic features of myelodysplastic syndrome and aplastic anemia in pediatric and young adult patients[J]. Haematologica.

[14] Brown AL, Hahn CN, Scott HS (2020). Secondary leukemia in patients with germline transcription factor mutations (RUNX1, GATA2, CEBPA)[J]. Blood.

[15] Greenberg PL, Attar E, Bennett JM, National Comprehensive Cancer Network (2011). NCCN Clinical Practice Guidelines in Oncology: myelodysplastic syndromes[J]. J Natl Compr Canc Netw.

[16] Cheloor Kovilakam S, Gu M, Dunn WG (2023). Prevalence and significance of DDX41 gene variants in the general population[J]. Blood.

[17] Polprasert C, Schulze I, Sekeres MA (2015). Inherited and Somatic Defects in DDX41 in Myeloid Neoplasms[J]. Cancer Cell.

[18] Quesada AE, Routbort MJ, DiNardo CD (2019). DDX41 mutations in myeloid neoplasms are associated with male gender, TP53 mutations and high-risk disease[J]. Am J Hematol.

[19] Lewinsohn M, Brown AL, Weinel LM (2016). Novel germ line DDX41 mutations define families with a lower age of MDS/AML onset and lymphoid malignancies[J]. Blood.

[20] Li P, Brown S, Williams M (2022). The genetic landscape of germline DDX41 variants predisposing to myeloid neoplasms[J]. Blood.

[21] Sébert M, Passet M, Raimbault A (2019). Germline DDX41 mutations define a significant entity within adult MDS/AML patients[J]. Blood.

[22] Takeda J, Yoshida K, Makishima H (2015). Genetic Predispositions to Myeloid Neoplasms Caused By Germline DDX41 Mutations[J]. Blood.

[23] Qu S, Li B, Qin T (2021). Molecular and clinical features of myeloid neoplasms with somatic DDX41 mutations[J]. Br J Haematol.

[24] Li P, White T, Xie W (2022). AML with germline DDX41 variants is a clinicopathologically distinct entity with an indolent clinical course and favorable outcome[J]. Leukemia.

[25] Nannya Y, Tobiasson M, Sato S (2023). Postazacitidine clone size predicts long-term outcome of patients with myelodysplastic syndromes and related myeloid neoplasms[J]. Blood Adv.

[26] Makishima H, Saiki R, Nannya Y (2023). Germ line DDX41 mutations define a unique subtype of myeloid neoplasms[J]. Blood.

[27] Arber DA, Orazi A, Hasserjian RP (2022). International Consensus Classification of Myeloid Neoplasms and Acute Leukemias: integrating morphologic, clinical, and genomic data[J]. Blood.

[28] Döhner H, Wei AH, Appelbaum FR (2022). Diagnosis and management of AML in adults: 2022 recommendations from an international expert panel on behalf of the ELN[J]. Blood.

[29] Baliakas P, Tesi B, Cammenga J (2024). How to manage patients with germline DDX41 variants: Recommendations from the Nordic working group on germline predisposition for myeloid neoplasms[J]. Hemasphere.

[30] Bougeard G, Renaux-Petel M, Flaman JM (2015). Revisiting Li-Fraumeni Syndrome From TP53 Mutation Carriers[J]. J Clin Oncol.

